# A Prospective Pilot Study to Validate the Management Protocol for Patients Presenting with Acute Urinary Retention: A Community-Based, Nonhospitalised Protocol

**DOI:** 10.1100/tsw.2006.379

**Published:** 2006-06-02

**Authors:** Shyamala S. Gopi, Chris M. Goodman, Allison Robertson, Derek J. Byrne

**Affiliations:** Department of Urology, Tayside University Hospitals NHS Trust, Ninewells Hospital, Dundee, UK

**Keywords:** acute painful urinary retention, urethral catheterisation, alpha blocker, community management of urinary retention

## Abstract

Acute urinary retention (AUR) in males is managed conventionally by hospital admission, alpha-adrenergic therapy, and trial without catheter. To reduce inpatient bed pressures, we set up a protocol to manage such patients in the community. We review our results in this paper. We performed a prospective study of male patients presenting to our acute admissions ward and Accident and Emergency department over 6 months. Patients with chronic urinary retention, macroscopic haematuria, sepsis, urinary tract infection, and/or serum creatinine >130 mmol/l were excluded from the study. Those enrolled were catheterised, commenced on alfuzosin (10 mg nocte), and discharged to the community. A trial without catheter (TWOC) was performed 5—7 days later. QoL/IPSS, peak flow rate, and residual volume assessment were performed following successful TWOC 3 months later.Thirty-one male patients with a median age of 69 years were studied and the median residual volume following catheterisation was 900 ml. The aetiology of AUR was benign prostatic hyperplasia (BPH) in 29 patients and constipation in the remaining 2 patients. TWOC was successful in 19 patients (61.3%) following first TWOC, 26 (83.9%) following second trial of voiding. The mean peak flow rate was 6.5 ml/sec and postvoid scan 165 ml, following an immediate TWOC. At 3 months follow-up, mean peak flow rate was 13.2 ml/sec, postvoid scan 26.5 ml, IPSS 4.5, and QoL score was 2. This study has shown that AUR can be managed safely and effectively in the community. Effective communication with the nurse urology specialist, general practitioner, and emergency department are crucial for the successful implementation of the protocol.

## INTRODUCTION

Benign prostatic hyperplasia (BPH) and lower urinary tract symptoms are common problems faced by aging men and affect up to 70% of men older than 60 years[1]. Clinical progression of BPH is defined by the development of acute urinary retention (AUR), renal insufficiency, recurrent urinary tract infection, incontinence, or an increase from baseline in the American Urological Association (AUA) symptom score index of 4 or more points. AUR is a common event in the natural history of BPH and may be the first manifestation of bladder outflow obstruction in up to 25%[1]. In Lytton's series of patients undergoing surgery, approximately 10–15% presented in acute retention[2]. AUR is both a significant public health issue and of consequence to the affected individual. The aetiology and pathogenesis of AUR are multifactorial. Several processes that are thought to initiate a sequence of events resulting in AUR include prostatic infarction, alpha-adrenergic overactivity, and neurotransmitter modulation[3]. Prostatitis, bladder overdistention, excessive fluid intake, medication, and debility have been identified as contributing factors[5]. Studies of the dynamics of AUR have shown that progressive stretching of smooth muscle fibre initially increases the contractile power and then, beyond a critical point, decreases it[4]. This is a known physiological property of smooth muscle. In AUR due to BPH, intravesical pressure falls and the pressure in the intraprostatic segment of the urethra is increased. Sympathetic activity is increased when overdistention of the bladder occurs. The administration of an alpha blocker reduces the pressure along the entire posterior urethra. Several studies have been published that emphasise the most effective way to treat AUR following initial catheterisation[5,6,10], but these patients have been managed for several decades as inpatients in many centres, even with the recent trend of the use of alpha-adrenergic blocker therapy followed by a trial without catheter (TWOC). The use of alpha blockers followed by successful TWOC as an inpatient has proven benefits in reducing peri- and postoperative morbidity and mortality[7,8].

Following a review of the pathophysiology of BPH and AUR, we set up a prospective study to manage male patients presenting to the Accident and Emergency department (A&E) and acute admissions unit with first-time AUR. Our goals were to manage such patients in the community safely and effectively, to reduce inpatient bed pressures, and to suggest that alpha-blocker therapy and catheterisation for a sufficient period could improve the successful outcome of TWOC.

## PATIENTS AND METHODS

We performed a prospective study of patients presenting with AUR to our A&E department and acute admissions ward between June 2004 and December 2004. They were managed according to a protocol devised by the authors (Fig. 1). Patients with chronic retention, macroscopic haematuria, sepsis, urinary tract infection, and abnormal serum creatinine (age/sex matched to the laboratory values) were excluded from the study. Those enrolled were catheterised, commenced on an alpha blocker (alfuzosin XL 10 mg nocte), and discharged home following an initial assessment including digital rectal examination (DRE) by the urologist on call. Verbal consent was obtained from all patients and they were evaluated by the nursing staff for safe discharge from the acute admissions unit or A&E. To ensure that the nurse practitioner and the general practioner (GP) received adequate information, the case notes from the A&E and the acute admissions ward were forwarded accordingly. The GP was advised to prescribe an alternative alpha blocker if the patient was intolerant to alfuzosin. The urology nurse specialist performed a TWOC 5–7 days later in the urology assessment unit. The successful outcome of TWOC was decided by an experienced urology nurse specialist who had obtained a degree in nursing/urological studies and was qualified by years of experience in conducting the nurse-led clinics for lower urinary tract symptoms. The patients who failed their first TWOC were either recatheterised or taught clean intermittent self-catheterisation (CISC) prior to the second trial of voiding. An appointment for the second trial of voiding was given in 2–4 weeks. Flow rate and postvoid residual volume assessment were performed following

## PROTOCOL FOR ACUTE URINARY RETENTION IN MEN

**FIRST-TIME ACUTE URINARY RETENTION IN MEN**

↓

Catheterise with 14–16 Ch 2 way urethral catheter. Note residual volume and DRE finding in case notes. If urine analysis positive, send for dip slide.

↓

**CRITERIA for Immediate Discharge**

Residual volume ≤1100 mls
No frank haematuria
White cell count <13, serum creatinine <120 mmol/dl (age/sex matched to lab values)
No signs of urosepsis
Clinically benign prostate gland.
Adequate dexterity and cognition/carer assistance if applicable

↓

Commence on **alpha blocker**, Tablet Alfuzosin XL 10 mg nocte if no contraindication. Explain side effects.
Laxatives if constipated.
Commence on Tablet Trimethoprim for 5 days if urine analysis is positive for nitrite and leucocytes.
To teach catheter management. Leaflet to be provided and supplies of catheter leg bag/valves and night bags
Discharge patient home with 2-weeks supply of alpha blocker.

↓

Inform district nurse and GP.
Ward or A&E to bleep urology nurse specialist between 9 am and 5 pm or fax details if referral is out of hours.
Appointment for trial of voiding to be organised between 5 and 7 days at nurse urology clinic.

↓

**Trial Without Catheter** followed by flow rate and postvoid scan.
Repeat DRE if necessary. Serum PSA to be requested if appropriate at a later date.

| **Successful** | **Unsuccessful** |
| --- | --- |
| Review at outpatient clinic in 3 months. Repeat flow test, postvoid scan, IPSS and QoL score. | To contact the on-call urology team for further advice and management. |

**FIGURE 1**

successful TWOC. Three months later, a repeat flow rate, postvoid scan, DRE, and Quality of Life (QoL)/International Prostate Symptom Score (IPSS) were performed. Serum prostate specific antigen (PSA) was measured, if indicated, at the 3 months follow-up. The urology team was contacted for further advice and management if the patient had an unsuccessful TWOC.

## RESULTS

This prospective study over a 6-month period involved 31 male patients.The aetiology of AUR was BPH in 29 patients and constipation in the remaining 2 patients (Table 1). Median age was 69 years (range 44–85 years). The median residual volume was 900 ml (range 300–1100 ml). Following commencement of alpha blocker (alfuzosin XL 10 mg nocte), TWOC was successful in 19 of the 31 patients (61.3%) following the first TWOC. The second flow test with a successful void rose to a further 26 of the 31 patients (83.8%) (Table 2).

**TABLE 1**
**Aetiology of AUR**

| Aetiology | Number of Patients |
|---|---|
| BPH | 29 |
| Constipation | 2 |

**TABLE 2**
**Immediate Outcome of TWOC**

| | |
|---|---|
| Postcatheterisation median volume (mls) at presentation | 900 (range 300–1100) |
| Successful TWOC (first and second) | 19/31 (61.3%); 26/31 (83.8%) |
| Median peak flow (Qmax in ml/sec) | 6.5 (range 3–25) |
| Postvoid residual (mls) | 165 (range 21–500) |
| Unsuccessful TWOC | 5 |

Following the first TWOC, median peak flow rate (Qmax) was 6.5 ml/sec (range 3–25); median postvoid residual was 165 ml (range 21–500 ml). At 3 months follow-up, IPSS was 4.5 (range 2–11), QoL was 2 (range 1–2), median peak flow increased to 13.2 (range 4–27), postvoid residual reduced to 26.5 mls (range 0–450) (Table 3).

**TABLE 3**
**3 Months Follow-Up Data of the 26 Patients Who Had a Successful Voiding**

| | |
|---|---|
| Peak flow Qmax in mls/sec | Median 13.2 (range 4–27) |
| Postvoid residual in mls | Median 26.5 (range 0–450) |
| IPSS | Median 4.5 (range 2–11) |
| QoL | Median 2 (range 1–2) |

Of the remaining five patients who were unable to have a successful void, one patient had significant comorbidity and patient preference resulted in long-term catheter, one patient preferred to be managed by CISC for incomplete bladder emptying, and three patients underwent bladder outlet surgery within 3 months.

None of the patients needed overnight hospital admission. Patient compliance with alpha blocker was good with no side effects reported. None of the patients encountered difficulty in managing the catheter and leg bag (see Tables 4 and 5).

**TABLE 4**
**Complications and Outcomes of Unsuccessful TWOC**

| | |
|---|---|
| Difficulty in managing leg bag | 0 |
| Number of readmissions | None |
| Side effect of alfuzosin | 0 |
| Long-term catheter | 1 |
| CISC | 1 |
| Bladder outlet surgery | 3 |

**TABLE 5**
**Unsuccessful TWOC in Relation to Age and Postcatheterisation Volume**

| Age in Years | Postcatheterisation Volume at Presentation (in mls) | Outcome of Unsuccessful TWOC |
|---|---|---|
| 83 | 1000 | TURP |
| 73 | 1000 | TURP |
| 66 | 500 | TURP |
| 65 | 1100 | CISC |
| 75 | 1100 | Long-term catheter |

## DISCUSSION

The paper focuses on managing AUR in the community. Studies have shown that catheter trial performed immediately had a success rate of 44%, 48 h later recorded a 51% success, and patients benefited the most with a TWOC after 1 week (62%). Younger age group, residual volume of less than 1 l, and increased period of drainage[6,7] were associated with a better outcome of TWOC. A long-term follow-up and benefits related to alpha blocker and TWOC has not been observed.

Our study showed that none of the patients needed overnight hospital admission. None came back for readmission with problems during the study period. The study highlights the importance of a multidisciplinary approach and effective communication with patient, district nurse, and GPs in helping to keep patients presenting with uncomplicated AUR out of the hospital. We followed a strict protocol in our study and with sufficient period of bladder drainage with alpha blocker, the percentage of successful TWOC has been encouraging. It also indicates that a second trial of voiding has proved beneficial for a few patients and the outcome has deferred or delayed surgery in 83.8% of the men who presented with AUR. This has facilitated preoperative assessment of the patient and thereby reduced the morbidity associated with the surgery performed on patients with indwelling catheter[8,9].

In conclusion, this study has shown that AUR can be managed in the community safely and effectively. The protocol has also paved the way to reduce inpatient bed pressure. The outcome of TWOC

following treatment with alpha blocker and sufficient period of catheterisation is merited in all patients presenting to A&E or acute admissions ward and discharged home with urethral catheter. The study also shows that a second trial of voiding has proved beneficial and is therefore worth considering in this category of men who have presented with AUR.

## References

1. Djavan, B., Madersbacher, S., Klingler, C., and Marberger, M. (1997) Urodynamic assessment of patients with acute urinary retention: is treatment failure after prostatectomy predictable? *J. Urol*. **158,** 1829–1833.
2. Lytton, B., Emery, J.M., and Harvard, B.M. (1968) The incidence of benign prostatic hypertrophy. *J. Urol*. **99**, 639–645.
3. Jacobsen, S.J., Jacobson, D.J., Girman, C.J., Roberts, R.O., Rhodes, T., Guess, H.A., and Lieber, M.M. (1997) Natural history of prostatism. Risk factors for acute urinary retention. *J. Urol*. 158, 481–487.
4. Lawson, J.D. and Tomlinson, W.B. (1952) Observations on the dynamics of acute urinary retention in man. *J. Urol*. **67,** 951–956.
5. Choong, S. and Emberton, M. (2000) Acute urinary retention. *BJU Int*. **85,** 185–201.
6. Taube, M. and Gajraj, H. (1989) Trial without catheter following AUR urine. *BJU* **63**, 180–182.
7. Djavan, B., Shariat, S., and Omar, M. (1998) Does prolonged catheter drainage improve the chance of recovering voluntary voiding after acute urinary retention (AUR)? *Eur. Urol*. **33,** 110.
8. Garibaldi, R.A., Burke, J.P., Dickman, M.L., and Smith, C.B. (1974) Factors predisposing to bacteriuria during indwelling urethral catheterisation. *N. Engl. J. Med*. **291**, 215–219.
9. ElMalik, E.M.A., Ibrahim, A.I.A., Gahli, A.M., Saad, M.S.M., and Bahar, Y.M. (2000) Risk factors in prostatectomy bleeding: preoperative urinary infection is the only reversible factor. *Eur. Urol*. **37**, 199–204.
10. McNeill, S.A., Daruwala, P.D., Mitchell, I.D.C., Shearer, M.G., and Hargreave, T.B. (1999) Sustained release of alfuzosin and trial without catheter after AUR. A prospective placebo controlled trial. *BJU* **84**, 622–627.
